# Prognostic Value of Perioperative Serum Creatinine Kinase Levels for Metachronous Colorectal Liver Metastasis

**DOI:** 10.7759/cureus.91753

**Published:** 2025-09-06

**Authors:** Yu Matsumoto, Yuichiro Otsuka, Hiroka Hosaka, Yoji Kajiwara, Rei Okada, Yuko Ito, Masaru Tsuchiya, Mitsunori Ushigome, Shuichiro Matoba, Hideaki Shimada

**Affiliations:** 1 Department of Surgery, Toho University School of Medicine, Tokyo, JPN; 2 Department of Surgery, Toho University Sakura Medical Center, Chiba, JPN; 3 Health Management Center, Japan Community Health Care Organization (JCHO) Funabashi Central Hospital, Chiba, JPN

**Keywords:** colorectal cancer, liver resection, metachronous liver metastasis, prognosis, serum creatine kinase

## Abstract

Background: Although decreased serum creatine kinase (s-CK) levels are observed in some cancers, possibly due to consumption during tumor growth, the relationship between s-CK levels and colorectal liver metastasis remains unclear. This study investigated the prognostic significance of perioperative s-CK levels in patients with metachronous colorectal liver metastasis.

Methods: A total of 46 patients who underwent liver resection from 2011 to 2023 were included in this study. The s-CK levels were assessed before liver resection and six months after liver resection. The patients were categorized into low or high s-CK groups, and the lower limit of normal s-CK levels was set as the cutoff value. The prognostic significance for recurrence-free survival (RFS) and overall survival (OS) was analyzed.

Results: There was no significant difference in RFS or OS between the preoperative low and preoperative high s-CK groups (P = 0.089, P = 0.132, respectively). There was a significant difference in RFS between the postoperative low and postoperative high s-CK groups (P = 0.001). However, there was no significant difference in OS between these groups (P = 0.072). Patients with consistently low s-CK levels before and six months after liver resection showed significantly poorer RFS (P = 0.002) and poorer OS (P = 0.038) compared with patients who had consistently high s-CK levels.

Conclusion: Perioperative s-CK levels may be useful as predictors of prognosis in patients with metachronous colorectal liver metastasis. Patients with consistently low perioperative s-CK levels may have a high risk for recurrence and mortality.

## Introduction

Approximately 30% of patients who undergo surgery for colorectal cancer subsequently experience metachronous colorectal liver metastasis [1]. Surgical resection of colorectal liver metastasis is an effective treatment for long-term survival, but the post-resection recurrence rate is high, ranging from 50%-70%, which is a problem [2]. Currently, serum carcinoembryonic antigen (CEA) and carbohydrate antigen 19-9 (CA19-9) are widely used as biomarkers for colorectal liver metastasis to assist in diagnosis, monitoring treatment response, and providing prognostic information [3,4]. The sensitivity and specificity of these markers have limitations, however, so new, complementary prognostic markers would be beneficial [5].

The enzyme serum creatine kinase (s-CK) is involved in energy metabolism across various tissues and reportedly has a role in cell division and the immune system [6,7]. Importantly, studies have indicated that low s-CK levels are a poor prognostic factor in colorectal cancer [8], hepatocellular carcinoma [9], esophageal cancer [10], gastric cancer [11], pancreatic cancer [12], breast cancer [13], and lung cancer [14]. Decreased s-CK levels may be associated with cancer progression, potentially linked to the increased consumption of energy substrates by rapidly proliferating cancer cells. The relationship between s-CK and colorectal liver metastasis remains unclear, and the role of perioperative changes in s-CK levels for predicting the prognosis of patients with metachronous colorectal liver metastasis has not been studied.

The objective of this study is to determine whether perioperative serum CK levels can serve as a prognostic marker in patients with resected metachronous colorectal liver metastases.

## Materials and methods

Patients

This retrospective study included 46 patients (31 male, 15 female) with a median age of 71 years (range: 38-85 years) who were diagnosed with metachronous colorectal liver metastasis and underwent radical liver resection between 2011 and 2023 at Toho University Omori Medical Center. This study was approved by the Ethics Committee of Toho University Omori Medical Center (#M24224 M23174 21320 21039 20200 20196 19056 18002) and conducted according to the guidelines of the Declaration of Helsinki. Information about the study was disclosed on the institution’s website, and potential participants were free to opt out. We accessed the medical records of the patients for this specific study in January 2025. The participants’ s-CK levels were measured at two time points: before and six months after liver resection. The associations of the clinicopathological factors of sex, age, body mass index, white blood cell counts, hemoglobin, albumin, CA19-9, and CEA with s-CK levels were evaluated. In this study, the lower limits of normal s-CK levels (males: 59 U/L, females: 41 U/L) were set as the cutoff values. The patients were categorized on the basis of this cutoff value into low and high s-CK groups to evaluate the association of s-CK with clinicopathological factors, recurrence-free survival (RFS), and overall survival (OS). RFS was defined as the interval from the date of surgery to the date of known recurrence, while OS was defined as the interval from the date of surgery to the date of death or last follow-up.

Statistical analysis

Fisher’s exact probability test and Student’s t‐test were performed for two-group comparisons. The Kaplan-Meier method was used to calculate RFS and OS, and the log-rank test was performed to evaluate differences between groups. Cox proportional hazards regression was used to perform multivariate analyses. EZR version 1.68 [15] was used to perform all statistical analyses. Values of two-sided P < 0.05 were accepted as indicating statistical significance.

## Results

Comparison of clinicopathological factors between the low and high s-CK groups before liver resection

In the low s-CK group, the number of females was significantly lower than the number of males (P = 0.018) (Table 1). Other factors, including age, body mass index, tumor size, white blood cell count, hemoglobin, platelet count, CA19-9, and CEA levels, were not significantly associated with the s-CK groups before liver resection.

**Table 1:** Comparison of clinicopathological factors between the low and high s-CK groups before liver resection *Fisher’s exact probability test

Variables		Number of patients (n = 46)	Serum creatine kinase before liver resection		P-value^*^
			Low group (n = 14)	High group (n = 32)	
Sex	Female	15	1	14	0.018
	Male	31	13	18	
Age (years)	<70	17	3	14	0.195
	≥70	29	11	18	
BMI (kg/m^2^)	<22	23	9	14	0.337
	≥22	23	5	18	
Maximum diameter of the tumor (mm)	<20	22	6	16	0.754
	≥20	24	8	16	
White blood cell (/μl)	<5500	21	7	14	0.755
	≥5500	25	7	18	
Hemoglobin (g/dl)	<12	14	7	7	0.084
	≥12	32	7	25	
Platelet (/μl)	<200000	20	7	13	0.748
	≥200000	26	7	19	
CA 19-9 (U/ml)	<40	40	10	30	0.060
	≥40	6	4	2	
CEA (U/ml)	<5	21	5	16	0.522
	≥5	25	9	16	

Prognostic significance of s-CK levels before liver resection

**Figure 1:**
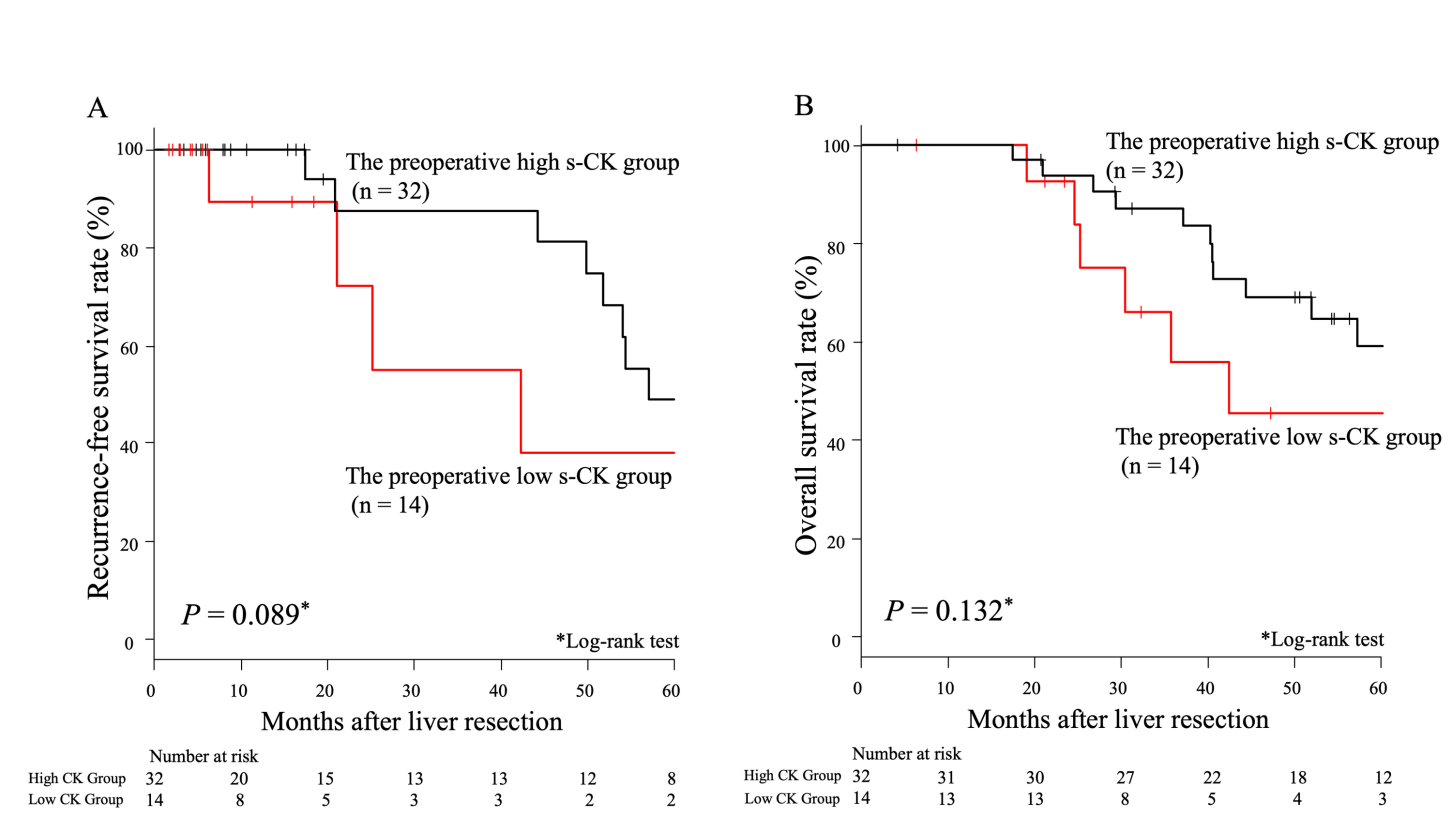
Comparisons of survival curves between the preoperative low and preoperative high s-CK groups A. Comparisons of recurrence‑free survival curves between the preoperative low and preoperative high s-CK groups B. Comparisons of overall survival curves between the preoperative low and preoperative high s-CK groups

There was no significant difference in RFS between the preoperative low and preoperative high s-CK groups (P = 0.089, Figure 1A). Similarly, there was no significant difference in OS between these groups (P = 0.132, Figure 1B). In the univariate analysis for RFS, low s-CK, high CA19-9 (≥40 U/ml), and high CEA (≥5 U/ml) were significantly associated with poor RFS (P = 0.001, P < 0.001, and P = 0.022, respectively) (Table 4, left panel). In the multivariate analysis for RFS, no independent predictor was associated with poor RFS (Table 2, right panel). 

**Table 2:** Univariate and multivariate analyses of clinicopathological factors predicting recurrence-free survival in patients before liver resection *Log-rank test, **Cox regression analysis

Variables		Number of patients (n = 46)	P-value*	Multivariate analysis hazard ratio (95% confidence interval)	P-value**
Sex	Male	15	0.208		
	Female	31			
Age (years)	≥70	17	0.374		
	<70	29			
BMI	≥22	23	0.911		
	<22	23			
Maximum diameter of the tumor (mm)	<20	22	0.585		
	≥20	24			
White blood cell (/μl)	<5500	21	0.636		
	≥5500	25			
Hemoglobin (g/dl)	<12	14	0.024	3.628 (0.778-16.920)	0.101
	≥12	32			
Platelet (/μl)	<200000	20	0.252		
	≥200000	26			
CA 19-9 (U/ml)	≥40	6	0.001	523800000 (0.000-Inf)	0.998
	<40	40			
CEA (U/ml)	≥5	25	0.585		
	<5	21			
Serum creatine kinase before liver resection (U/L)	Low group	14	0.089	1.875 (0.623-5.637)	0.508
	High group	32			

Comparison of clinicopathological factors between the low and high s-CK groups after liver resection

Tumors with a diameter ≥20 mm were observed more frequently in the low s-CK group than in the high s-CK group (P = 0.009) (Table 3).

**Table 3:** Comparison of clinicopathological factors between the low and high s-CK groups after liver resection *Fisher’s exact probability test

Variables		Number of patients (n = 46)	Serum creatine kinase before liver resection		P-value^*^
			Low group (n = 14)	High group (n = 32)	
Sex	Female	15	3	12	0.667
	Male	31	4	27	
Age (years)	<70	17	1	16	0.234
	≥70	29	6	23	
BMI (kg/m^2^)	<22	23	4	19	1
	≥22	23	3	20	
Maximum diameter of the tumor (mm)	<20	22	0	22	0.009
	≥20	24	7	17	
White blood cell (/μl)	<5500	25	3	22	1
	≥5500	21	4	17	
Hemoglobin (g/dl)	<12	16	4	12	0.216
	≥12	30	3	27	
Platelet (/μl)	<200000	25	3	22	0.686
	≥200000	21	4	17	
CA 19-9 (U/ml)	<40	41	5	36	0.160
	≥40	5	2	3	
CEA (U/ml)	<5	33	4	29	0.385
	≥5	13	3	10	

Prognostic significance of s-CK levels after liver resection

**Figure 2:**
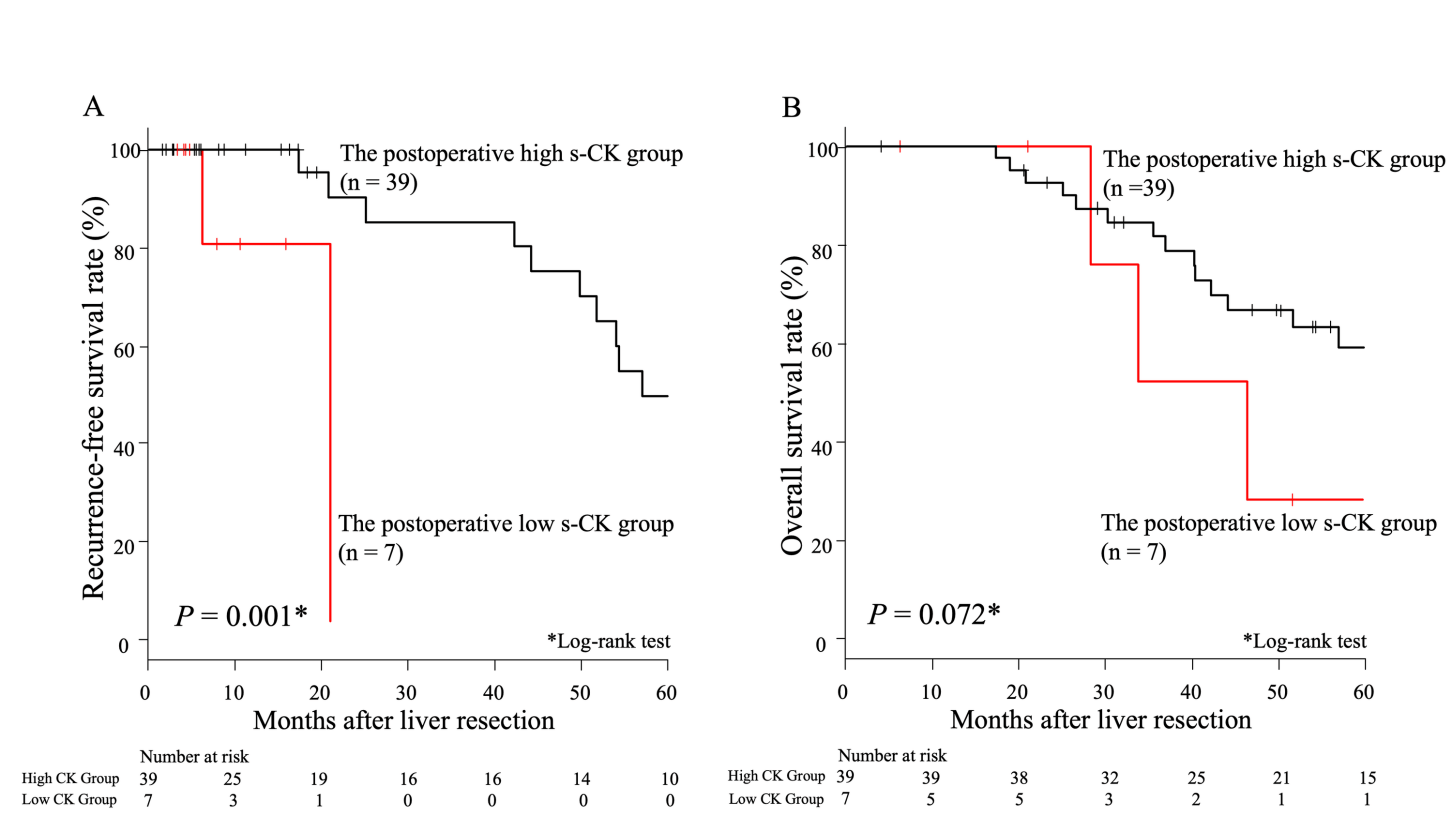
Comparisons of survival curves between the postoperative low and high s-CK groups A. Comparisons of recurrence‑free survival curves between the postoperative low and high s-CK groups B. Comparisons of overall survival curves between the postoperative low and high s-CK groups

There was a significant difference in RFS between the postoperative low s-CK and postoperative high s-CK groups (P = 0.001, Figure 2A). However, there was no significant difference in OS between these groups (P = 0.072, Figure 2B). In the univariate analysis for RFS, low s-CK, high CA19-9 (≥40 U/ml), and high CEA (≥5 U/ml) were significantly associated with poor RFS (P < 0.001, P = 0.022, and P = 0.001, respectively) (Table 4, left panel). In the multivariate analysis for RFS, no independent predictor was associated with poor RFS (Table 4, right panel).

**Table 4:** Univariate and multivariate analyses of clinicopathological factors predicting recurrence-free survival in patients after liver resection *Log-rank test, **Cox regression analysis

Variables		Number of patients (n = 46)	P-value*	Multivariate analysis Hazard ratio (95% confidence interval)	P-value**
Sex	Male	15	0.208		
	Female	31			
Age (years)	≥70	17	0.374		
	<70	29			
BMI	≥22	23	0.911		
	<22	23			
Maximum diameter of the tumor (mm)	<20	22	0.585		
	≥20	24			
White blood cell (/μl)	<5500	25	0.678		
	≥5500	21			
Hemoglobin (g/dl)	<12	16	0.108	1.918 (0.482-7.634)	0.356
	≥12	30			
Platelet (/μl)	<200000	25	0.979		
	≥200000	21			
CA 19-9 (U/ml)	≥40	5	<0.001	161400000 (0.000-Inf)	0.998
	<40	41			
CEA (U/ml)	≥5	13	0.022	2.340(0.533-10.270)	0.256
	<5	33			
Serum creatine kinase before liver resection (U/L)	Low group	7	0.001	5.396 (0.356-81.800)	0.224
	High group	39			

Perioperative changes in s-CK levels

**Figure 3:**
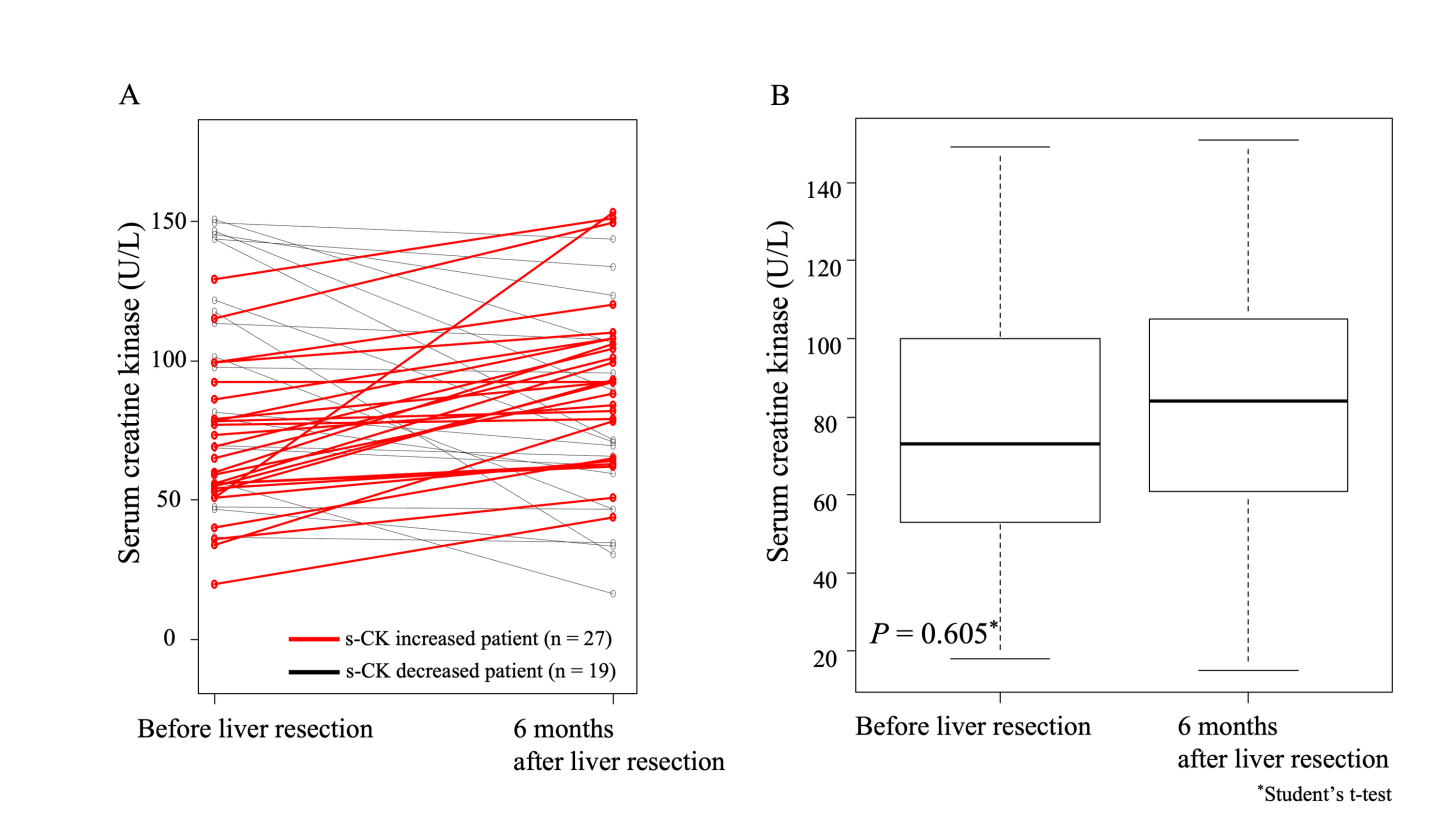
Perioperative changes in s-CK levels A. The perioperative changes in s-CK levels in individual patients B. Comparison of the mean s-CK levels between before and six months after liver resection

Figure 3A shows the perioperative changes in s-CK levels in individual patients. The s-CK levels increased in 27 patients and decreased in 19. Figure 3B compares the mean s-CK levels between before and six months after liver resection. The comparison showed no significant difference in the mean s-CK levels before and after liver resection (P = 0.605).

Effect of the changing patterns of s-CK levels on prognosis

**Figure 4:**
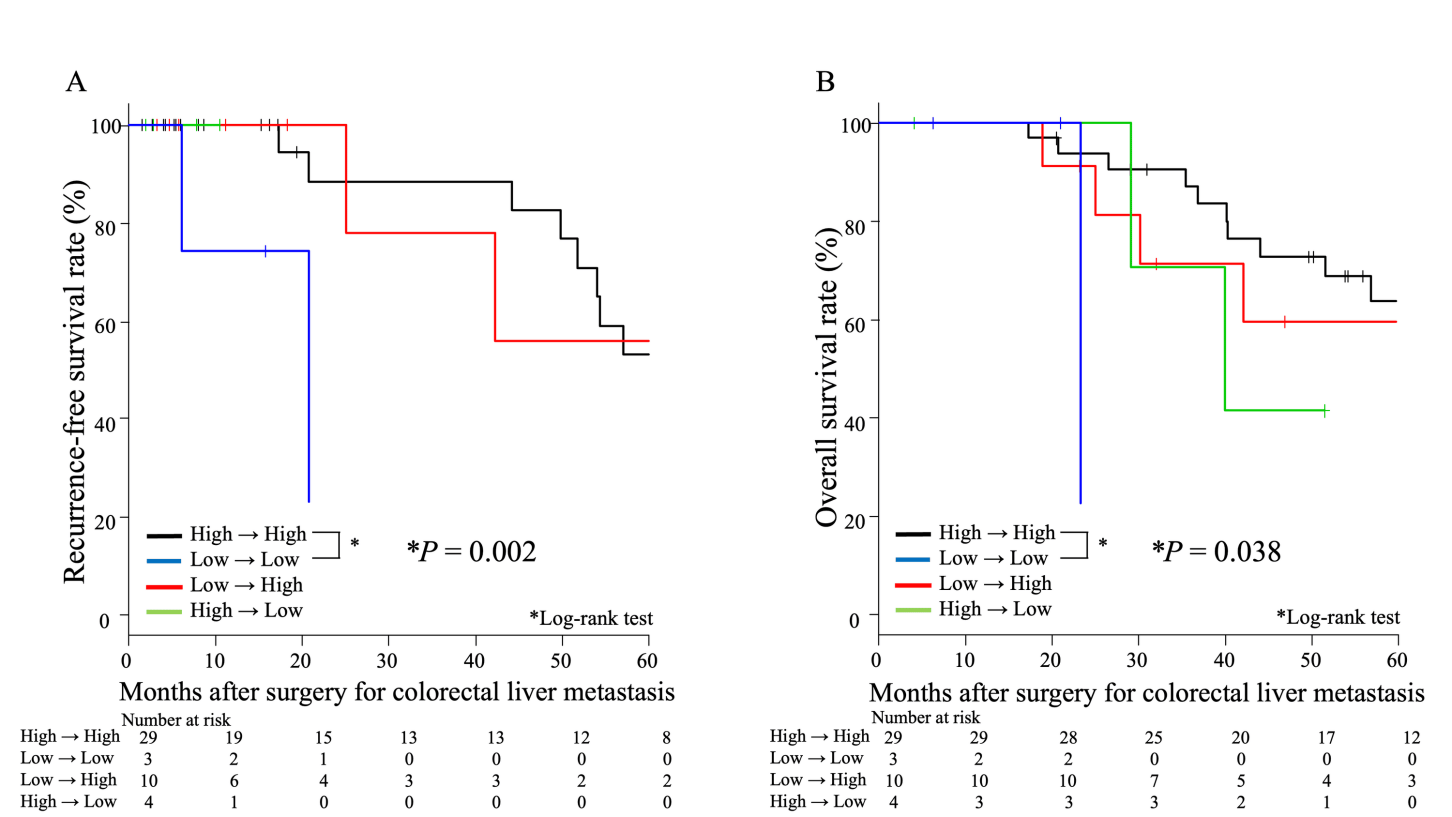
Effect of the changing patterns of s-CK levels on prognosis A. Comparisons of recurrence‑free survival curves based on s-CK status before liver resection and six months after liver resection B. Comparisons of overall survival curves based on s-CK status before liver resection and six months after liver resection

Figure 4 shows survival outcomes based on the perioperative changes in s-CK levels. The patients were divided into four groups according to their changing patterns of s-CK levels (classified based on specific cutoff values as “low” or “high”): High→High (n = 29), Low→Low (n = 3), Low→High (n = 10), and High→Low (n = 4). There was a significant difference in RFS between the consistently low s-CK (Low→Low group) and consistently high s-CK groups (High→High group) (P = 0.002, Figure 4A). There was also a significant difference in OS between the consistently low s-CK (Low→Low group) and consistently high s-CK groups (High→High group) (P = 0.038, Figure 4B).

## Discussion

We investigated the prognostic significance of perioperative s-CK level changes in patients with metachronous colorectal liver metastasis. We found that the perioperative patterns of s-CK-level change were significantly associated with both RFS and OS in patients who underwent liver resection for colorectal liver metastasis. Notably, patients who exhibited consistently low s-CK levels both before and after liver resection showed significantly poor outcomes.

To our knowledge, this is the first report to analyze the relationship between s-CK levels after cancer resection and postoperative prognosis. Large tumors were associated with the postoperative low s-CK group. Although there appeared to be no correlation with prognosis, s-CK levels may remain lower after liver resection of large tumors than after liver resection of small tumors. Creatine, which is related to energy production, is synthesized in the liver [7]. Since the larger the volume of liver metastases, the greater the volume of liver resection, the ability to synthesize creatine may be reduced after liver resection. During the postoperative period, as the liver regenerates, creatine levels decrease, so the production of s-CK, which consumes creatine and converts it into energy, may also decrease.

Among postoperative clinicopathological factors for colorectal liver metastasis in RFS, low hemoglobin levels, high CA19-9 levels, and high CEA levels have been reported as independent poor prognostic factors [16,17]. In our study, low hemoglobin levels, high CA19-9 levels, and high CEA levels showed significant differences in the univariate analysis that did not remain after the multivariate analysis. The lack of independence in the multivariate analysis may be due to the following: first, the sample size, with only 46 cases, is limited; second, hemoglobin, CA19-9, and CEA are probably confounded with s-CK.

In the present study, we hypothesized that preoperative s-CK levels decrease due to cancer proliferation-related consumption and increase after liver resection because the cancer cells are removed. Although there was no statistically significant difference between the median s-CK levels before and after liver resection, the number of patients in which the postoperative s-CK levels increased relative to the preoperative s-CK levels was greater than the number of patients in which the postoperative s-CK levels decreased relative to the preoperative s-CK levels. This finding supports our hypothesis concerning perioperative changing patterns, as the consistently low s-CK group had significantly worse RFS and OS than the consistently high s-CK group, despite the small sample sizes. All three patients in the consistently low s-CK group experienced recurrence and death within two years. Consistently low s-CK levels are associated with poor prognosis, possibly due to cancer-cell proliferation and reduced antitumor immunity. Active cancer cells require energy for rapid proliferation. Therefore, we assume cancer cells obtain energy by consuming creatine kinase and converting adenosine triphosphate to adenosine diphosphate. Conversely, T-cells possess antitumor immune functions through creatine metabolism and have an essential role in defending against cancer cells [7]. In a tumor state, cancer cells may use creatine kinase in the blood more than T-cells, resulting in decreased T-cell production and weakened antitumor immunity. To our knowledge, no reported studies have compared preoperative and postoperative s-CK levels in malignant tumors. Our study is the first attempt to investigate the effect of s-CK level changes before and after liver resection on prognosis in patients with metachronous colorectal liver metastasis. A novel finding, even in this small study, is that the changes in s-CK levels, particularly consistently low s-CK levels, were associated with poor prognosis.

This study had several limitations. First, the study sample size was small (n = 46). This is a major limitation since it significantly reduced the statistical power of the study. Second, the s-CK has three isoenzymes: CK-MM, CK-MB, and CK-BB. In pancreatic cancer, it has been suggested that analyzing the ratio of these s-CK isoenzymes to the total s-CK could provide a more accurate assessment of the role of s-CK as a prognostic factor [18]. Unfortunately, because this was a retrospective study, it was not possible to measure all three isoenzyme levels. Since this was a single-center retrospective study, we plan to address these limitations in future research by conducting a multicenter study with a much larger patient sample.

## Conclusions

In conclusion, this study investigated the prognostic significance of perioperative s-CK level changes in patients undergoing resection for metachronous colorectal liver metastasis. Our findings suggest that preoperative s-CK levels alone cannot predict outcomes, but that dynamic changes in s-CK levels over time may serve as a potential prognostic marker. Specifically, patients who exhibited consistently low s-CK levels both before and after liver resection (the Low→Low group) had significantly worse RFS and OS. This suggests that monitoring perioperative s-CK, a simple and widely available blood test, could be an effective tool for post-surgical risk assessment. Due to limitations such as the small sample size, these findings need validation through larger, multicenter, prospective studies to confirm the role of s-CK in clinical practice.
